# Tensin4 (TNS4) is upregulated by Wnt signalling in adenomas in multiple intestinal neoplasia (Min) mice

**DOI:** 10.1111/iep.12352

**Published:** 2020-06-22

**Authors:** Teresa P. Raposo, Abdulaziz Alfahed, Abdolrahman S. Nateri, Mohammad Ilyas

**Affiliations:** ^1^ Division of Cancer and Stem Cells School of Medicine University of Nottingham Nottingham UK; ^2^ Nottingham Molecular Pathology Node University of Nottingham Nottingham UK; ^3^ Department of Medical Laboratory College of Applied Medical Sciences Prince Sattam Bin Abdulaziz University Al‐Kharj Saudi Arabia; ^4^ Cancer Genetics and Stem Cell Group, Cancer Biology Division of Cancer and Stem Cells School of Medicine University of Nottingham Nottingham UK

**Keywords:** *Apc*^Min/+^, HCT116, multiple intestinal neoplasia, TNS4, Wnt signalling

## Abstract

*Apc* ^Min/+^ mice are regarded as a standard animal model of colorectal cancer (CRC). *Tensin4 (TNS4 or Cten)* is a putative oncogene conferring features of stemness and promoting motility. Our objective was to assess TNS4 expression in intestinal adenomas and determine whether TNS4 is upregulated by Wnt signalling. Apc^Min/+^ mice (n = 11) were sacrificed at approximately 120 days old at the onset of anaemia signs. Small intestines were harvested, and Swiss roll preparations were tested for TNS4 expression by immunohistochemistry (IHC). Individual polyps were also separately collected (n = 14) and tested for TNS4 mRNA expression and *Kras* mutation. The relationship between Wnt signalling and TNS4 expression was tested by Western blotting in the human CRC cell line HCT116 after inhibition of β‐catenin activity with MSAB or its increase by transfection with a Flag β‐catenin expression vector. Overall, 135/148 (91.2%) of the total intestinal polyps were positive for TNS4 expression by IHC, whilst adjacent normal areas were negative. RT‐qPCR analysis showed approximately 5‐fold upregulation of TNS4 mRNA in the polyps compared to adjacent normal tissue and no *Kras* mutations were detected. In HCT116, β‐catenin inhibition resulted in reduced TNS4 expression, and conversely, β‐catenin overexpression resulted in increased TNS4 expression. In conclusion, this is the first report linking aberrant Wnt signalling to upregulation of TNS4 both during initiation of intestinal adenomas in mice and in in vitro models. The exact contribution of TNS4 to adenoma development remains to be investigated, but the *Apc* ^Min/+^ mouse represents a good model to study this.

## 1 INTRODUCTION

C57BL/6J *Apc* ^Min/+^ mouse strain [also known as multiple intestinal neoplasia (Min) mice] constitutes a standard experimental model of colorectal cancer, mimicking the familial adenomatous polyposis (FAP) disease in humans.^1^ This strain has a transversion point mutation on nucleotide 2549 T/A, which converts *Apc* codon 850 from leucine to a stop codon, resulting in a truncated protein. Heterozygous *Apc* ^Min/+^ mice are viable and quickly develop abundant intestinal adenomas (predominantly in the small intestine) due to the loss of the wild‐type *Apc* allele. The loss of wild‐type APC protein results in a failure to control levels of β‐catenin protein, thereby resulting in constitutive activation of Wnt signalling.^1^, ^2^, ^3^ This, in turn, leads to the initiation of the adenomas.

TNS4 is an oncogene overexpressed in a variety of human tumours including colorectal cancer (CRC).^4^, ^5^, ^6^ We have previously shown that it is expressed in large human adenomas and that it is positively regulated by *Kras* signalling.^7^ We have also shown that TNS4 can confer a number of properties such as stemness and epithelial‐mesenchymal transition (EMT), which can contribute to cancer hallmarks. Whilst the latter would be relevant to invasion and metastasis, stemness would be important in early tumour development. Since aberrant activation of Wnt signalling promotes stemness and leads to the development of an adenoma (ie the first step in the development of CRC), we hypothesized that TNS4 could be a target of Wnt signalling and thereby mediate some of the stemness‐inducing activity of Wnt signalling. In this study, we sought to test our hypothesis in vivo by assessing the expression of TNS4 in intestinal adenomas in *Apc* ^Min/+^ mice and in vitro by looking at the expression of TNS4 following modulation of Wnt signalling in the human CRC cell line HCT116.

## 2 METHODS

### 2.1 Breeding and genotyping

C57BL/6J‐*Apc^Min/+^* mice were obtained from Jackson Laboratories and bred to wild‐type C57BL/6J females at the BSU facility, Medical School, University of Nottingham. Genotyping was performed using an in‐house method for PCR of an 85‐bp amplicon followed by high‐resolution melting (HRM) analysis to determine T/A heterozygous substitution. The PCR contained 250nM primers (Forward: GGG AAG TTT AGA CAG TTC TCG T; Reverse: TGT TGG ATG GTA AGC ACT GAG) with 1× HotShot Diamond Master mix (Clent Life Science) and 5% Eva Green. PCR was performed in a PeqLab gradient PCR thermocycler, and the cycling conditions were as follows: denaturation at 95°C, 5 minutes, 40 cycles of 95°C for 40 seconds, 58.5°C for 20 seconds, 72°C for 20 seconds and finally 72°C, 5 minutes, 95°C, 2 minutes and storage at 4°C. After the reaction, 10 µL of the PCR product was transferred to a 96‐well black hardshell plate (Bio‐Rad laboratories, HSP9661) and overlaid with 20 µL mineral oil (Sigma‐Aldrich, M5904). After a short centrifugation for 5 minutes at 1750 xg, the PCR products were melted on a lightscanner (Idaho technologies, Biofire Defense) preheated to 62°C. Wild‐type control samples were set as a baseline, representing negative controls without mutations on the regions analysed, and *Apc^Min/+^* could be detected by a left shift in the melting curve, caused by unstable heteroduplex formation in *Apc^Min/+^* mutants.

### 2.2 Ethical approval

Ethical approval for this project was granted by the Bio‐Support Unit, University of Nottingham.

### 2.3 Tissue processing, polyp counts and immunohistochemistry

A total of 11 *Apc^Min/+^* mice were humanely sacrificed between 120 and 140 days of age, at the onset of anaemia signs, by CO_2_ sedation followed by cervical dislocation. The whole intestine was collected, washed, opened and flattened using a *gut‐roller* device as previously described.^8^ The small intestinal segments were divided into thirds (SB1 = anterior third, SB2 = medial third, SB3 = posterior third), whilst the large intestine was kept whole. The intestines were fixed in neutral buffered formalin for 2‐3 hours, briefly stained with 0.2% methylene blue and then washed in PBS. As there were very few or no polyps in the large intestine (<1 polyp per mouse), only polyps arising in the small intestine were evaluated. Polyps were counted at 3x magnification with a dissecting microscope as previously described.^9^ The intestines were then fixed in formalin and embedded in paraffin as Swiss roll preparations.^10^

Immunohistochemical staining of TNS4 was performed on 4‐µm microtome sections. These were deparaffinized with xylene and then rehydrated through graded alcohols. Citrate buffer (pH 6.0) heat‐induced antigen retrieval was performed for 20 minutes, at 800 W power in a microwave and followed by endogenous peroxidase blocking with 0.3% hydrogen peroxide for 10 minutes and protein blocking for 5 minutes (Novolink Polymer Detection Kit). Rabbit anti‐human TNS4 antibody (clone SP83, Thermo Fisher) was incubated at a 1:100 dilution in 1% BSA in TBS, overnight, 4°C, followed by incubation with goat anti‐rabbit HRP secondary antibody (Sigma‐Aldrich) diluted to 1:200, for 1 hour at room temperature. Detection of hybridized antibody was performed by incubation with DAB for 5 minutes. The slides were digitized at 20× magnification using the Roche DP200 Ventana slide scanner and images analysed for positivity with Qupath version 0.1.2. Adenomas were manually annotated, and analysis was performed automatically by choosing positive cell detection analysis in optical density sum images and scoring the cytoplasm optical density mean. TNS4 positivity was considered if a polyp expressed more than 0.25% TNS4‐positive cells, the same level found in a wild‐type mouse small bowel. As a negative control, we have used duplicate sections where the primary antibody was replaced by its diluent (1% BSA in TBS). Colonic swiss rolls were used as a positive control as well as portions of pancreatic tissue attached to the duodenum on the first, most anterior small bowel segment.

### 2.4 RNA extraction and RT‐qPCR

Tissue samples from polyps (n = 14), adjacent normal intestine (n = 12) and normal intestine from wild‐type controls (N = 3) were also collected into RNA later (Sigma‐Aldrich). Reverse‐transcription quantitative PCR (RT‐qPCR) was performed to quantify relative gene expression of TNS4 using TBP as a housekeeping gene. Briefly, total RNA was extracted from homogenized tissue (MP Biomedicals homogenizer) with a GenElute mammalian RNA kit (Sigma‐Aldrich). Of this, 1 µg was converted to cDNA by using M‐MLV‐RT (Promega) according to the manufacturer's instructions. The qPCR was performed using GoTaq qPCR master mix with SYBR green, 250 nmol/L primers (TNS4 FWD: TGTTTGGAAGCAATCAGTCCCT; TNS4 RVS: TACTAGGAGCCTGGGCATCA; TBP FWD: GAAGAACAATCCAGACTAGCAGCA; TBP RVS: CCTTATAGGGAACTTCACATCACAG) and 40 ng of cDNA input on a Stratagene MX3005 qPCR thermocycler. The cycling conditions included 95°C denaturation for 2 minutes, 40 cycles of 95°C for 30 seconds, 60°C for 60 seconds and 72°C for 30 seconds. This was followed by a melting step at 95°C for 60 seconds, 55°C for 30 seconds and 95°C for 30 seconds.

Screening for *Kras* mutation was performed on cDNA, by PCR‐HRM on a lightscanner (Idaho). Briefly, a PCR to amplify exon 2 of *Kras* (the mutation hot spot) was set up with 40 ng of cDNA template, 1x Hot shot diamond master mix (Clent Life Science), 5% Eva Green (Biotium), 250 nmol/L primers (Forward primer: GCCTGCTGAAAATGACTGAGT; Reverse primer: CTCTATCGTAGGGTCATACTCATCC). PCRs were set up in triplicates for each biological sample and run on a PeqLab thermocycler with the same conditions as for the APC mutant allele detection, except annealing temperature which was 60°C.

### 2.5 Cell culture and Wnt‐signalling inhibition

Human colorectal adenocarcinoma HCT116 cell line was obtained from the ATCC and authenticated by SRT profiling. Cells were maintained in DMEM, 10% FBS, 4 mmol/L L‐glutamine (Gibco) at 37°C, 5%CO_2_ and regularly passaged or fed every 3‐4 days. To test for the effect of Wnt‐signalling inhibition on TNS4 expression, cells were treated for 24 hours with MSAB ((Methyl 3‐(4‐methylbenzenesulfonamido) benzoate, Sigma‐Aldrich), a specific β‐catenin inhibitor, at 3 and 6 µmol/L concentration with DMSO used as control. Overexpression of β‐catenin was achieved with transfection of a FLAG‐β catenin expression construct. Each well on a 6‐well plate was seeded with 10^6^ HCT116 cells, and total of 5 µg plasmid per well was transfected using lipofectamine 2000 in accordance with the manufacturer's instructions.

### 2.6 Protein extraction and Western Blot

Protein lysates were obtained by incubating cells with ice‐cold RIPA buffer supplemented with 1% proteinase phosphatase inhibitor (Pierce). Lysates were centrifuged at 16 000 *g* for 30 minutes, 4°C, the supernatant was collected, and protein was quantified by the BCA assay (Thermo Fisher Scientific). For Western blotting, 40 µg of protein was loaded on a Bis‐Tris 4%‐12% Acrylamide Novex Gel (Invitrogen), separated by electrophoresis at 150 V, 90 minutes, and then transferred for 30 minutes onto a PVDF membrane on a semi‐dry transfer system (Turboblot, Bio‐Rad laboratories). After brief Ponceau Red staining, the membrane was blocked for 1 hour at room temperature with 5% Skimmed Milk (Sigma‐Aldrich) in TBST (Tris‐buffered Saline Tween‐20, 0.1%) and the following antibodies incubated overnight, at 4°C; mouse anti‐human TNS4 clone 3B8 (Sigma‐Aldrich) diluted 1:1000, mouse anti‐human alpha‐tubulin clone DM1A (Abcam) diluted 1:5000, Active; rabbit anti‐human non‐phosphorylated beta‐catenin (CST) diluted 1:1000; and anti‐FLAG diluted 1:1000 followed by the respective HRP‐conjugated secondary antibody at appropriate dilution. Immunoblots were incubated with ECL (Amersham HE), and chemiluminescence was detected with a Li‐cor Odyssey scanner.

## 3 RESULTS AND DISCUSSION

In the macroscopic count, an average of 42.14 ± 16.68 polyps per mouse (N = 11) could be observed.

**FIGURE 1:**
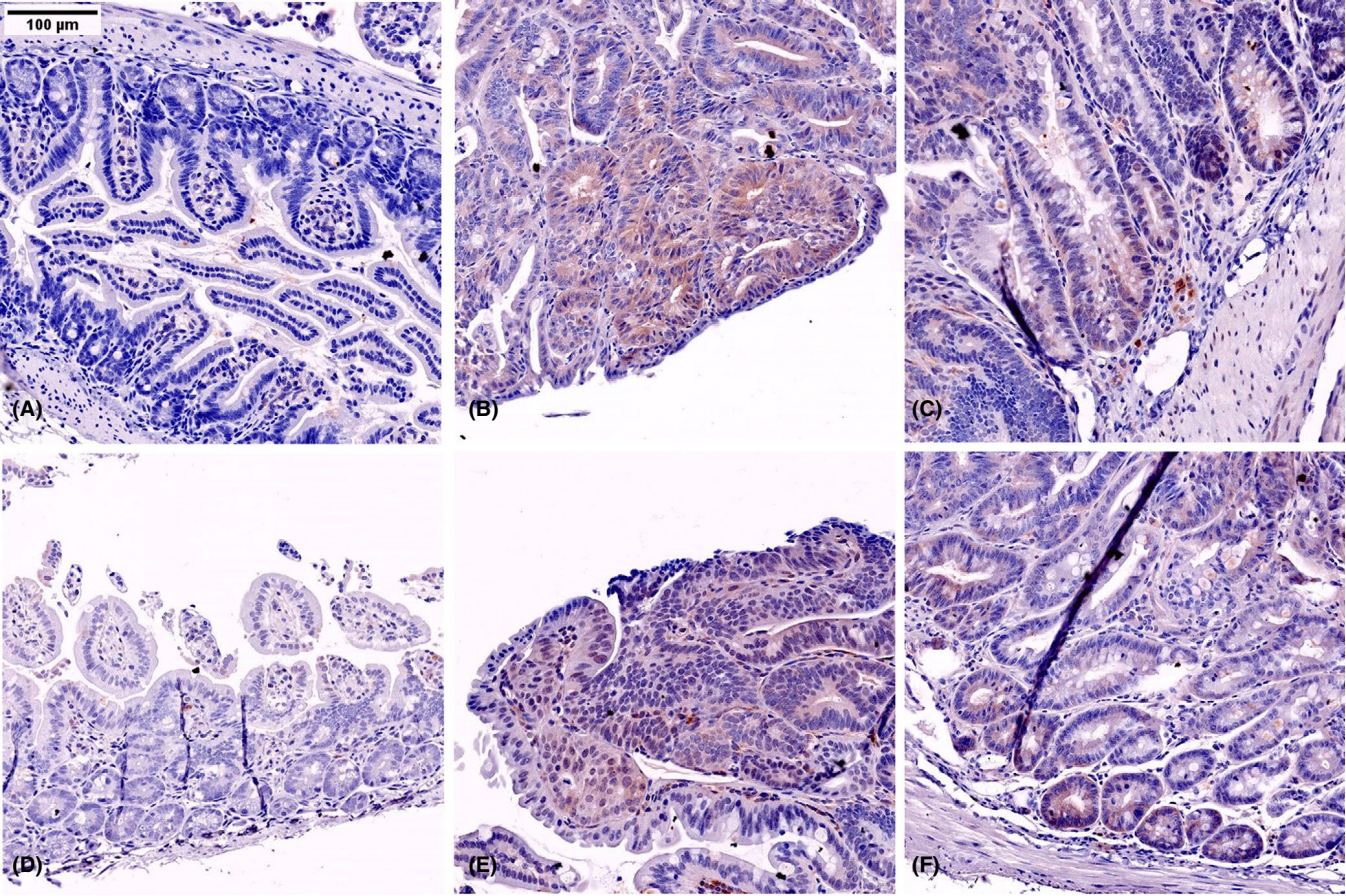
TNS4 protein expression detected by immunohistochemistry in the Apcmin polyps (B, C, E, F). Adjacent normal areas are negative in APCmin (A) and WT (D)

In the tissue sections, normal areas of the small bowel in between polyps did not show any significant expression of TNS4 (Figure 1). A total of 148 adenomas were visible in the Swiss rolls, and 135 (91.2%) were positive with no variation in expression seen between the polyps arising the three segments of the small bowel (Table S1 and Figure S2). In order to evaluate the level of change of TNS4 expression in the adenomas of *Apc* ^Min/+^ mice, levels of mRNA expression were quantified using RT‐qPCR in a total of 14 polyps. A comparative analysis of adenoma and adjacent normal tissue was performed using the ΔΔCt method. This showed approximately 5‐fold upregulation of TNS4 in the polyps compared to adjacent normal tissue.

**FIGURE 2:**
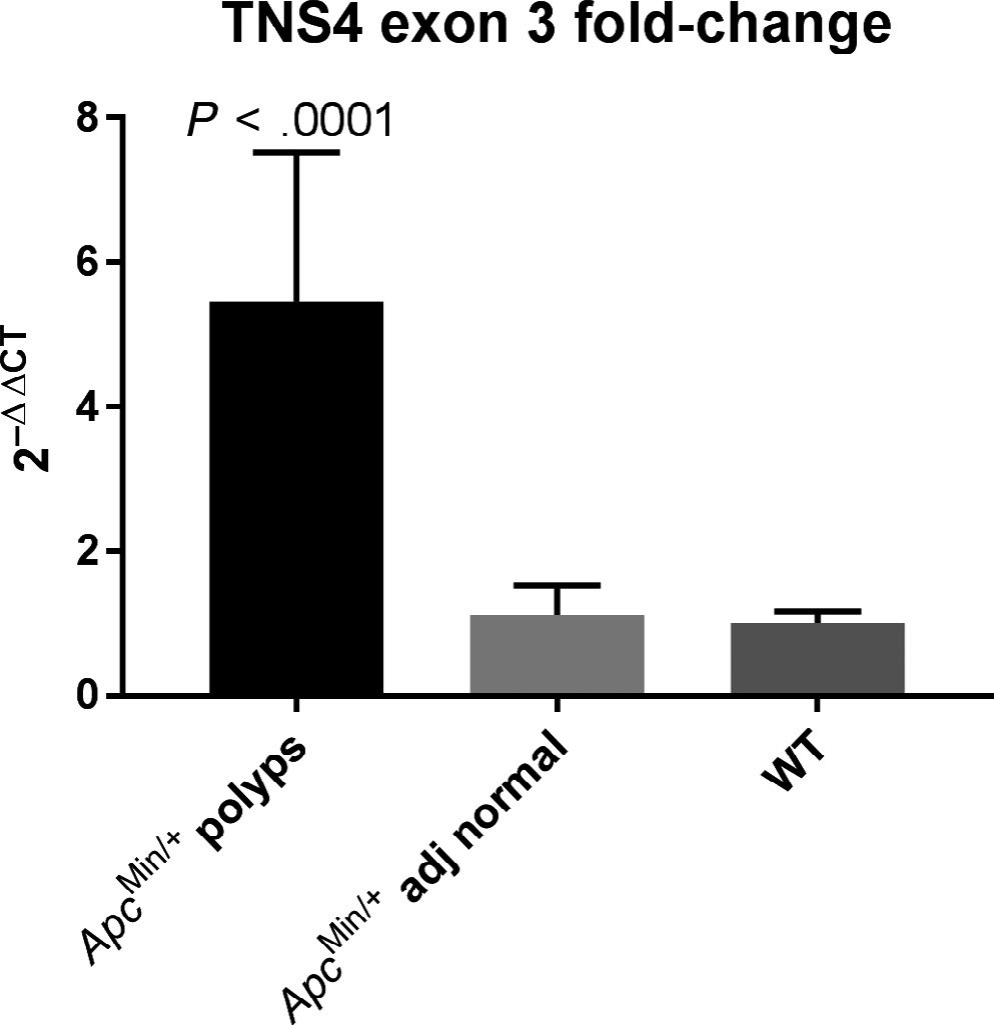
RT‐qPCR analysis of TNS4 exon 3 fold‐change in ApcMin/+ polyps and adjacent normal tissue relatively to WT C57BL6/J mice intestine. Mann‐Whitney U test was performed, and a 2‐tailed (exact) *P*‐value is shown

**FIGURE 3:**
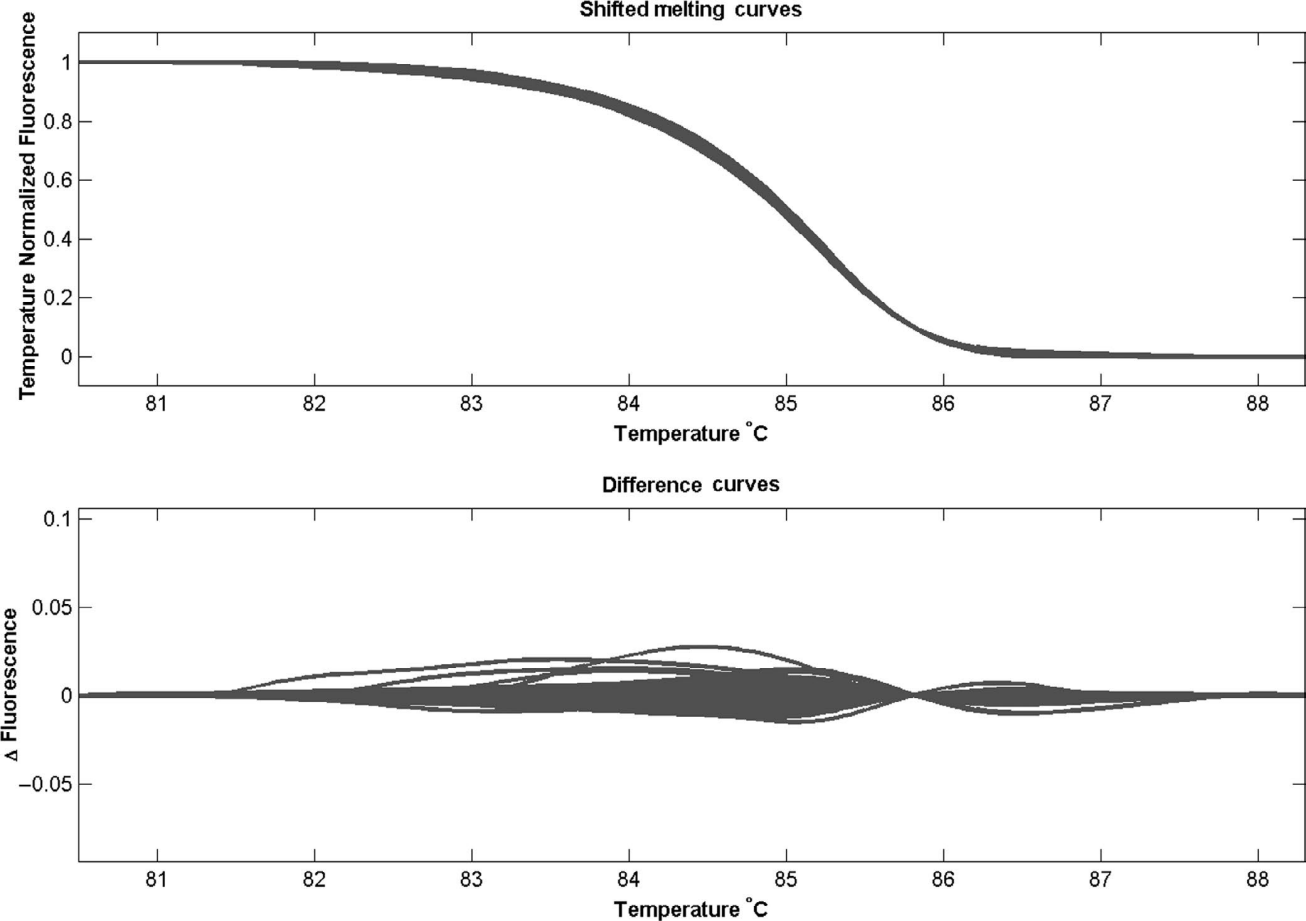
Mouse Kras exon 2 melting curves showing no variation amongst the polyps cDNA, adjacent normal tissue control and wild‐type controls

Our data show unequivocally that TNS4 is upregulated in the adenomas of *Apc* ^Min/+^ mice. The normal tissue of Min mice showed similar levels of TNS4 expression to that seen in the intestine of wild‐type mice (Figure 2). This implies that the TNS4 upregulation seen in these adenomas could be secondary to the event that causes adenoma development, that is activation of Wnt signalling. We have previously shown that TNS4 is a target of Ras‐Raf signalling. Even though it has long been known that adenomas in *Apc* ^Min/+^ mice never acquire *Kras* mutation, we felt it was important to exclude the possibility of *Kras* mutation as a cause of TNS4 upregulation in these polyps. We tested for *Kras* mutation hot spot on exon 2 by PCR‐HRM. We found no mutations in either the *Apc* ^Min/+^ adenomas or the normal tissue (Figure 3), thus supporting the possibility that activation of Wnt signalling was responsible for upregulation of TNS4.

**FIGURE 4:**
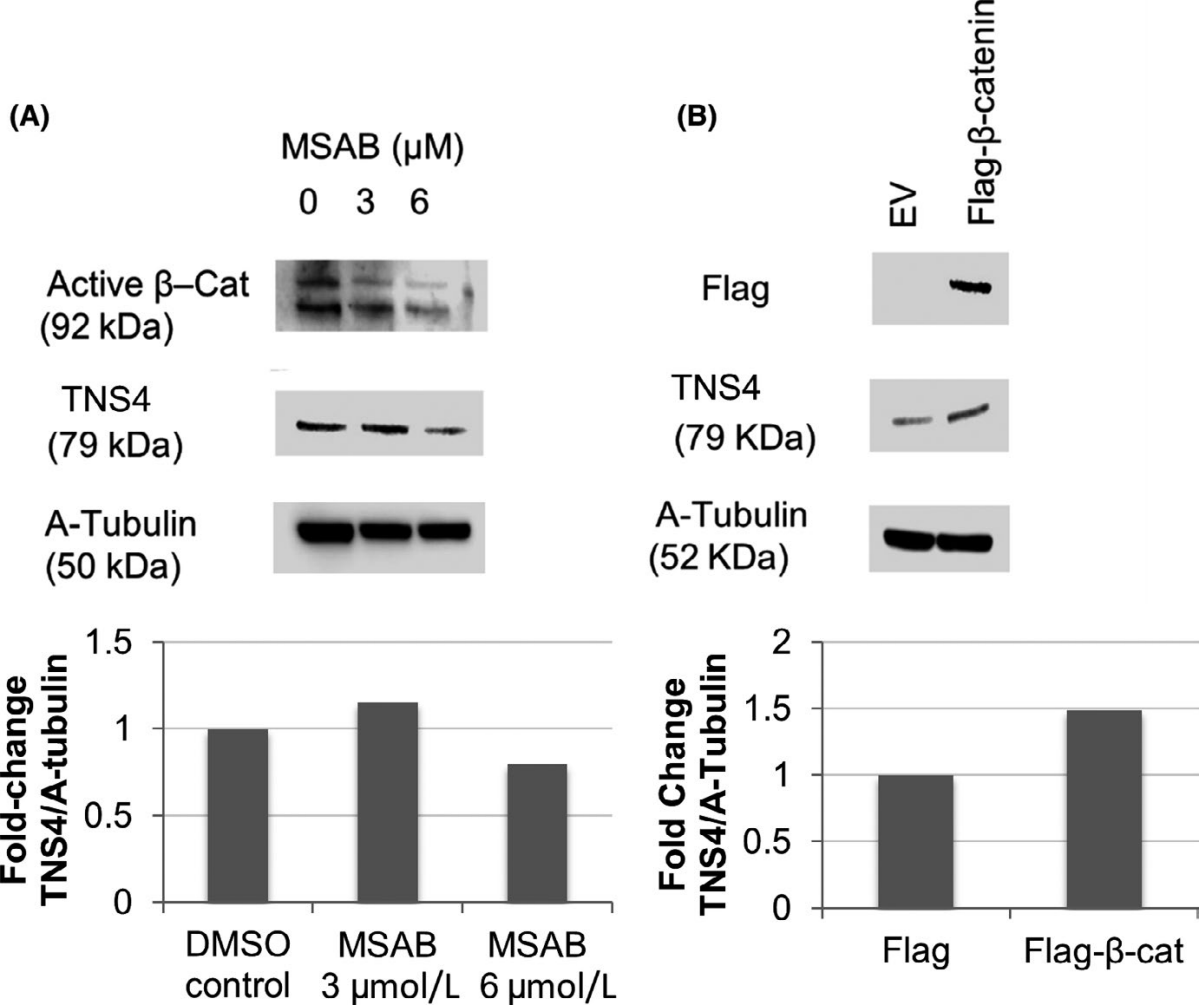
In HCT116 cells, inhibition of beta‐catenin following treatment with MSAB at 0‐6 umol/L caused a decrease in TNS4 (Cten) expression (A). By overexpressing beta‐catenin using a Flag‐tagged vector, Cten expression was upregulated (B)

As with all experiments performed in *Apc* ^Min/+^ mice, our data carry two caveats; that is, the observations are made in mice and the polyps are arising in the small intestine. To further investigate the relationship between Wnt‐signalling and TNS4 expression, we modulated levels of β‐catenin in a human CRC cell line (HCT116). This cell line is wild type for the *APC* gene but has a heterozygous β‐catenin mutation (deleted S45), and it expresses low levels of TNS4.^11^, ^12^ β‐Catenin is the mediator of canonical Wnt signalling, and levels of active β‐catenin can be reduced using the inhibitor MSAB. This is a cell‐permeable compound that binds to β‐catenin and induces its ubiquitination and proteasomal degradation. When HCT116 cells were treated with MSAB, there was a reduction in active β‐catenin and TNS4 levels were also downregulated (Figure 4A). To validate this and exclude the possibility of off‐target effects, we performed the reverse experiment and modulated active β‐catenin upwards. We ectopically expressed a FLAG‐tagged β‐catenin^13^ and this was followed by upregulation of TNS4 expression (Figure 4B), thereby confirming that TNS4 can be regulated by Wnt‐signalling activation.

This is the first report showing that TNS4 is upregulated at the very first step of intestinal tumorigenesis, that is the conversion of the normal epithelium into an adenoma. This is also the first report linking the upregulation of TNS4 with aberrant Wnt signalling. The precise mechanism by which Wnt signalling induces TNS4 expression is uncertain, but since TNS4 has previously been found in complex with β‐catenin,^14^ it is possible that there may also be some protein stabilization. Here, we have shown that adenomas in *Apc* ^Min/+^ mice show an average of a 5‐fold increase in TNS4 mRNA,thus, in this model at least there is transcriptional activation. Even though no putative transcription binding sites were listed when the human TNS4 promoter region was identified,^15^ we have conducted a search using Tfsitescan^16^ and Promo,^17^ which has identified likely binding sites for TCF4/LEF1 and C‐myc, which could be the responsible transcription factors (Figure S1).

The exact role of TNS4 plays in the development of early adenomas, which is uncertain. We and others have previously shown that TNS4 can alter several different biological processes resulting in a reduction of cell adhesion, epithelial‐mesenchymal transition (EMT) and promotion of stemness.^12^, ^18^, ^19^ Of these, the latter activity is most likely to give the greatest selective advantage in adenomas, and we have shown that TNS4 can promote stemness through interaction with Src by increasing colony‐forming ability.^20^ Since Wnt signalling is generally accepted as a promoter of stemness in the intestine (and other tissues), the easiest inference is that TNS4 mediates some of the stemness‐promoting activity of Wnt signalling in adenomas.

It has previously been shown that TNS4 is positively regulated by Ras‐Raf‐MAPK signalling,^21^, ^22^ and thus, it is activated by at least two signalling pathways. However, we and others have shown that TNS4 is also the target of several growth factors signalling pathways (such as epidermal growth factor, transforming growth factor β and fibroblast growth factor).^18^, ^20^, ^23^ Given the multiple roles of TNS4, it may be at the centre of a variety of different signalling pathways regulating important functions. Also, the role of TNS4 may change with gene dosage as tumours develop. Therefore, it may be activated by Wnt signalling at the initiation of adenomas where it may have a role in tumour establishment. In more advanced adenomas which acquire *Kras* mutation, TNS4 gene dosage may change as well as its role in tumour development.

In conclusion, to the best of our knowledge, this is the first report of TNS4 overexpression in intestinal polyps of *Apc* ^Min/+^ mice and its regulation by Wnt signalling. Previously, TNS4 has been shown to bind to beta‐catenin in the nucleus^19^ and suggested to promote colonic tumorigenicity via beta‐catenin, although a signalling mechanism was not proposed. TNS4 expression in *Apc* ^Min/+^ polyps also confirms previous results obtained in cases of human FAP patients^6^ and validates the suitability of this mouse model for further investigation of the role of TNS4 in colorectal carcinogenesis. By using HCT116, we have shown that TNS4 acts as a target of Wnt signalling and it is transcriptionally upregulated during the initiation of adenomas. The nature of its role is uncertain, but it may be related to the promotion of stemness. Taken together with published literature, TNS4 seems to be upregulated by multiple signalling pathways, thereby underlining its importance in cancer biology.^23^

## CONFLICT OF INTEREST

The authors declare no conflict of interest.

## Supporting information

Figure S1.

Figure S2.

Table S1.
