# Supplementary figures and images for: Tensin4 (TNS4) is upregulated by Wnt signalling in adenomas in multiple intestinal neoplasia (Min) mice
Source: Int J Exp Pathol. 2020 Jun 22;101(3-4):80–6. doi: 10.1111/iep.12352 (PMC7370848; doi:10.1111/iep.12352)

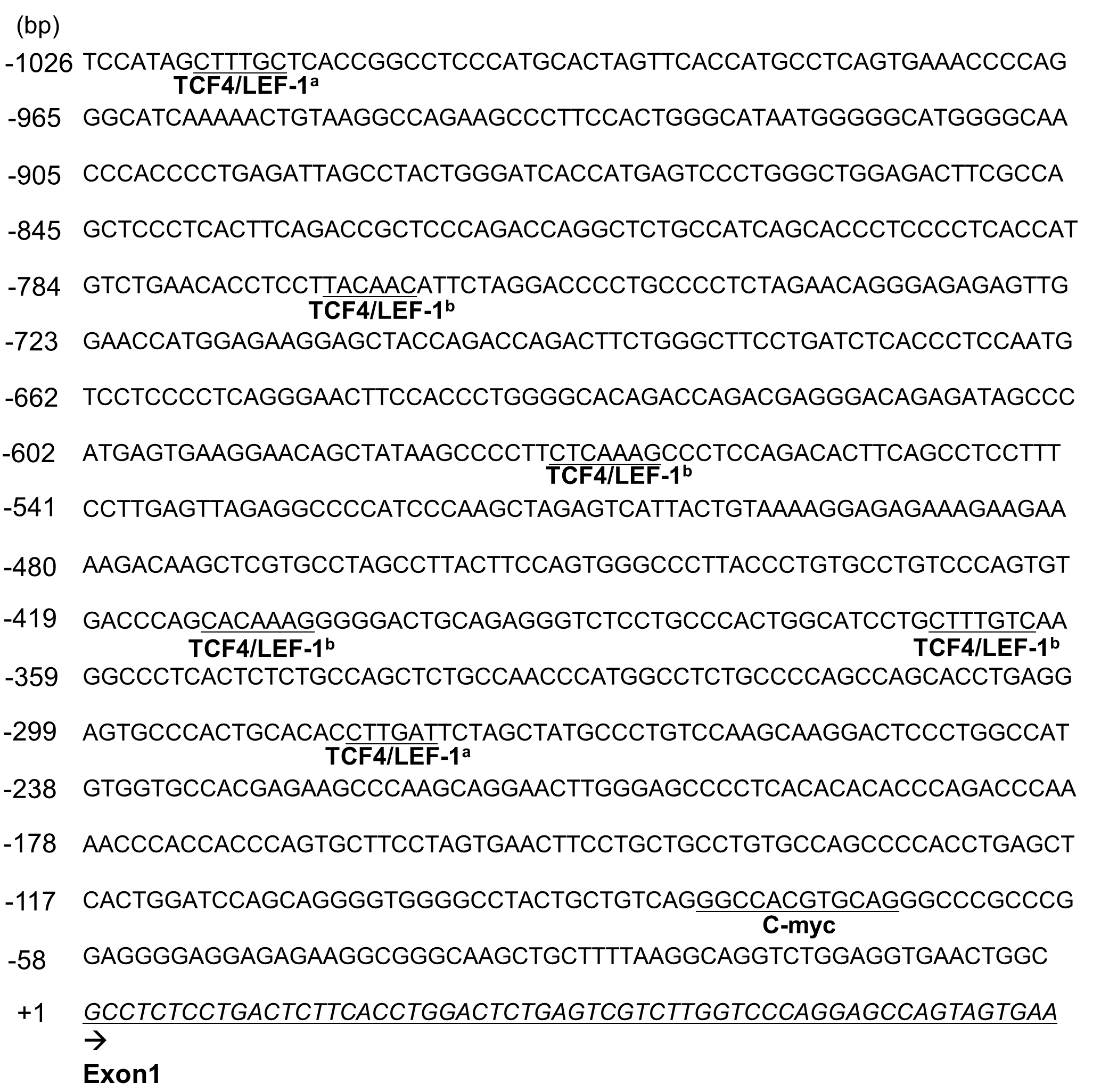

Supplement: Supplementary file 1 — Figure S1. [file IEP-101-80-s001.tif]

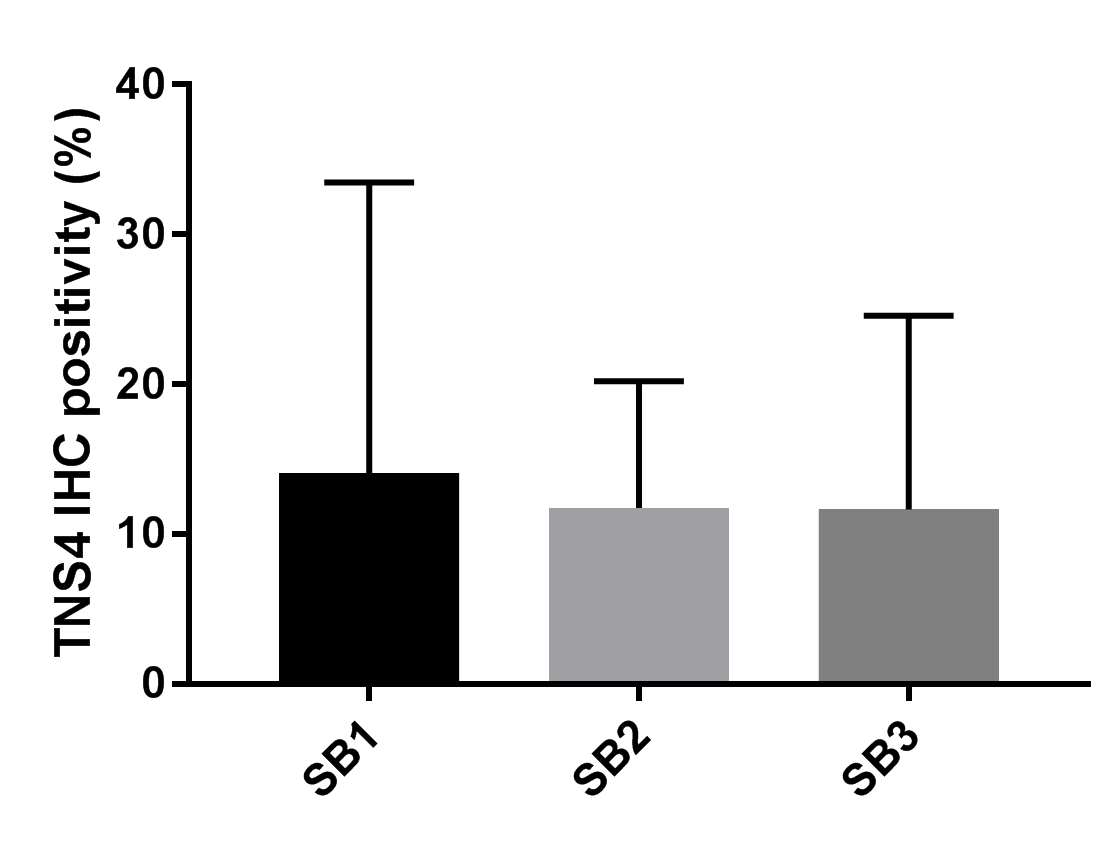

Supplement: Supplementary file 2 — Figure S2. [file IEP-101-80-s002.tif]
