# Supplementary material for: Tensin4 (TNS4) is upregulated by Wnt signalling in adenomas in multiple intestinal neoplasia (Min) mice
Source: Int J Exp Pathol. 2020 Jun 22;101(3-4):80–6. doi: 10.1111/iep.12352 (PMC7370848; doi:10.1111/iep.12352)
Supplement: Supplementary file 3 — Table S1. [file IEP-101-80-s003.docx]

|  | Mean TNS4 IHC positivity per polyp (%) | Standard deviation |
| --- | --- | --- |
| SB1 | 14.08 | 19.41 |
| SB2 | 11.75 | 8.45 |
| SB3 | 11.64 | 12.94 |
| Total mean SB | 12.23 | 12.54 |
| Maximum | 70.83 |  |
| Minimum | 0.00 |  |

Supplementary Table 1 – Mean TNS4 positivity (%) and standard deviation per polyp according to each of the small bowel segment (SB1 – anterior, SB2 – medial, SB3 – distal), as well as maximum and minimum values of TNS4 expression found in a polyp.
